# Population Structure of *Listeria monocytogenes* in Emilia-Romagna (Italy) and Implications on Whole Genome Sequencing Surveillance of Listeriosis

**DOI:** 10.3389/fpubh.2020.519293

**Published:** 2020-09-18

**Authors:** Erika Scaltriti, Luca Bolzoni, Caterina Vocale, Marina Morganti, Ilaria Menozzi, Maria Carla Re, Stefano Pongolini

**Affiliations:** ^1^Risk Analysis and Genomic Epidemiology Unit, Istituto Zooprofilattico Sperimentale della Lombardia e dell'Emilia-Romagna, Parma, Italy; ^2^Operating Unit of Clinical Microbiology, Regional Reference Center for Microbiological Emergencies, St. Orsola-Malpighi Polyclinic, Bologna, Italy

**Keywords:** *Listeria monocytogenes*, genomic epidemiology, surveilance, whole genome sequencing, cgMLST, SNPs, relatedness threshold, interpretation

## Abstract

The population structure of human isolates of *Listeria monocytogenes* in Emilia-Romagna, Italy, from 2012 to 2018 was investigated with the aim of evaluating the presence of genomic clusters indicative of possible outbreaks, the proportion of cluster-associated vs. sporadic isolates and different methods and metrics of genomic analysis for use in routine surveillance. In the 2012–2018 period the notification rate of confirmed invasive cases in Emilia-Romagna was 0.91 per 100,000 population per year, more than twice the average rate of EU countries. Out of the total 283 cases, 268 (about 95%) isolates were typed through whole genome sequencing (WGS) for cluster detection with methods based on core-genome multi-locus sequence typing and single nucleotide polymorphisms. Between 66 and 72% of listeriosis cases belonged to genomic clusters which included up to 27 cases and lasted up to 5 years. This proportion of cluster-associated cases is higher than previously estimated in other European studies. Rarefaction analysis, performed by reducing both the number of consecutive years of surveillance considered and the proportion of isolates included in the analysis, suggested that the observed high proportion of cluster-associated cases can be ascribed to the long surveillance duration (7 years) and the high notification and typing rates of this study. Our findings show that a long temporal perspective and high surveillance intensity, intended as both exhaustiveness of the system to report cases and high WGS-typing rate, are critical for sensitive detection of possible outbreaks within a WGS-based surveillance of listeriosis. Furthermore, the power and complexity of WGS interpretation emerged from the integration of genomic and epidemiological information in the investigation of few past outbreaks within the study, indicating that the use of multiple approaches, including the analysis of the accessory genome, is needed to accurately elucidate the population dynamics of *Listeria monocytogenes*.

## Introduction

*Listeria monocytogenes* is the causative agent of listeriosis, a severe food-borne disease mainly observed among elderly people, immunocompromised individuals, pregnant women, and newborn. The disease is relatively rare, but its high hospitalization and fatality rates and increasing incidence make it one of the most important food-borne infections in Europe and worldwide (1).

The confirmed identification of outbreaks and their sources of contamination is generally hampered by the long incubation of listeriosis and the common finding of *L. monocytogenes* in the food-chain and the environment. Consequently, this is only possible when laboratory surveillance is implemented and high-resolution typing methods, able to pinpoint similar isolates in the population and in the sources of infection, are used. For this reason, whole genome sequencing (WGS), a highly discriminatory method, is being progressively introduced in routine surveillance of listeriosis and in food-safety monitoring (2). However, the complexity of WGS data not only brings the potential of high-resolution molecular epidemiology, but also entails the need for robust and shared methods and standards for the generation and interpretation of typing results. In recent years different methods and interpretation criteria for WGS surveillance of *L. monocytogenes* have been developed from collections of isolates of various origin (3–8). This study analyzed the use of WGS for epidemiological surveillance of *L. monocytogenes* in the real-life conditions of the actual surveillance system of a specific administrative territory and explored its implications on surveillance strategies. The study territory is the Italian administrative Region of Emilia-Romagna (ER) having a population of about 4.5 million and a surface of about 22,450 Km^2^. Since 2012, the Region has implemented laboratory surveillance of food-borne diseases integrated with official microbiological monitoring of foodstuff and represents a typical European example of regional health administration. Emilia-Romagna adopted PCR-serogrouping as the routine typing method for *L. monocytogenes* isolates until 2017, while adopted WGS as the routine typing method starting from 2018. In the 2012–2018 period the notification rate of confirmed invasive cases, here defined as the number of confirmed invasive cases per 100,000 population [as used in (9, 10)], was 0.91 per 100,000 per year, more than twice the average rate of EU countries (9, 10). Despite the high notification rate, unexpectedly, no listeriosis outbreaks were detected in Emilia-Romagna before of the implementation of WGS surveillance. In this work, a retrospective (2012–2017) and prospective (2018) study of the population structure of human *L. monocytogenes* from ER was conducted to support the adoption of functional interpretation criteria for WGS surveillance. The aims of the study were: (i) to assess the presence of clusters of isolates similar enough to represent possible outbreaks, considering that before WGS typing was introduced all notified cases had been classified as sporadic and no outbreaks had been reported in ER; (ii) to determine the structure of the population of *L. monocytogenes*, namely the proportion of isolates belonging to clusters vs. the proportion of sporadic isolates, as an indicator of the prevalent mode of infection (outbreak-associated vs. sporadic) and the duration and geographical extension of clusters; (iii) to create a background of genomes of *L. monocytogenes* from the Regional territory to allow for accurate assessment of genomic relationships among the incoming isolates in the new surveillance; and (iv) to evaluate methods and metrics of genomic analysis for their use in the surveillance.

## Materials and Methods

### Bacterial Isolates, Whole Genome Sequencing, and Archive Genomes

The study included all 283 *L. monocytogenes* isolates notified to the laboratory surveillance of Emilia-Romagna (Italy) in the 2012–2018 period. All isolates originated from invasive cases and were checked to avoid any duplication (i.e., multiple isolates for single patient). The average notification rate for confirmed cases in Emilia-Romagna in the study period was 0.91 per 100,000 population per year, more than three times the overall national rate of 0.25 per 100,000 population per year (9, 10) and higher than those estimated in all but two EU countries in the same period, i.e., Finland and Denmark (9, 10). A total of 268 of the isolates (about 95%) underwent WGS, for the others, WGS was not possible (isolate not viable) or associated metadata were missing. Additionally, four environmental isolates that originated from outbreak investigations performed during the perspective surveillance underwent WGS. Genomic DNA was extracted with DNeasy Blood and Tissue kit (Qiagen), sequencing libraries were prepared using Nextera DNA Flex Library Prep Kit (Illumina) and run with a Miseq sequencer (Illumina) in pair-end mode (2 × 250 bp). The raw reads of the 272 newly sequenced isolates of the study were deposited at EBI under Project number PRJEB34036 (see Table S1). The raw reads of all available Italian genomes of human isolates, isolated in the years 2012–2018, were recovered from Sequence Reads Archive of NCBI [see (11–14)]. Table S2 provides a complete list of the accession numbers of the NCBI raw reads used.

### Core-Genome Multi-Locus Sequence Typing and SNP Analysis

Raw reads were checked for quality and length with FastQC (15) and for species confirmation using the miniKraken database (16), then filtered with Trimmomatic ver. 0.38 (17) according to (8), i.e., removal of (a) any adaptor sequences, (b) leading bases with PHRED <25, (c) trailing bases with PHRED <25, and (d) the entire read if length <36 bases, clipping of the remainder of the read when a sliding window of 20 bases has average PHRED <25. *De-novo* assembly was done with SPAdes Assembler ver. 3.9.0 (18) as in Van Walle et al. (8) and evaluated by QUAST ver. 4.2 (19) retaining high quality assemblies, i.e., post-trimming coverage > 40X, N50>20,000 Kb, (4), minimum contig length of 300 nucleotides (8), and contig number <50. Based on assemblies, phylogenetic lineage, clonal complex (CC), seven-loci Multi-Locus Sequence Types (ST) (20) were assigned through Bacterial Isolate Genome Sequence Database (BIGSdb), and core-genome Multi-Locus Sequence Types (cgMLST) were assigned through Bionumerics Software ver. 7.6.3 (Applied-Maths, Biomerieux) according to the Pasteur cgMLST scheme (4). Isolates with more than one locus showing multiple alleles and/or <95% of core genome coverage were re-processed, in accordance with Van Wall et al. (8). Cluster analysis based on cgMLST was done with single-linkage clustering. The metadata (Accession and sample numbers, source of origin, isolation material, date of isolation, age) and the main genomic clustering data (lineage, CC, ST) on the newly sequenced isolates were summarized in Table S1.

Matrices of Single Nucleotide Polymorphisms (SNP) were generated by CFSAN Pipeline for SNP analysis ver. 0.8.2 (FDA, USA) (21) and used to infer phylogeny within each ST through Maximum Likelihood Algorithm in RaxML software (22). The presence in ST7 isolates of the 31 kb prophage reported in 2015TE24968 genome (13) was checked by reads mapping using Geneious software (Biomatters, Ltd).

### Criteria for Cluster Definition

Clusters of similar isolates representing putative outbreaks of infection were defined according to different internationally proposed thresholds and criteria. We defined clusters as groups of at least two isolates similar according to specific allelic/SNPs thresholds [as in (8, 23)] and we classified as sporadic the isolates not belonging to any cluster. In the study, clusters of similar isolates were only considered as indicators of possible outbreaks while a confirmed outbreak is such only following identification of a source of infection common to the involved cases. Two cluster-defining thresholds were represented by different values of the maximum acceptable number of differing cgMLST loci between any pairs of genomes in a cluster. This number is commonly referred to as allelic distance (AD). The threshold values for AD adopted in this study were 7 and 4, hereafter referred to as AD7 and AD4, as already proposed by Moura et al. (4) and Van Walle et al. (8), respectively. In other words, isolates were considered part of the same cluster when their pairwise AD was ≤ 7 or ≤ 4 in cgMLST. The third threshold considered was the value of 5 as the maximum acceptable number of pairwise SNPs between any pairs of genomes in the same cluster, proposed by Dallman et al. (24), hereafter referred to as SNP5. The fourth approach adopted for cluster definition was that proposed by Pightling et al. (6), consisting in the combination of the following three criteria: (i) pairwise SNP distance <21; (ii) bootstrap support > 0.89 for the cluster clade in the SNP-based phylogenetic tree; (iii) monophyletic topology of the cluster isolates in a SNP-based phylogenetic tree, hereafter referred to as SBT (i.e., SNPs, Bootstrap, Topology).

### Rarefaction Analysis

The variation of the overall proportion of isolates attributed to clusters as a function of (a) the number of consecutive years of surveillance considered in the analysis and of (b) different fractions of isolates included in the analysis was estimated (rarefaction analysis). Cluster detection in rarefaction analysis was based on cgMLST with thresholds AD7 and AD4. Different rarefaction curves were generated corresponding to proportions of included isolates of 25, 50, and 100%. Estimations for all rarefaction curves were performed with 1,000 random samples of the isolates dataset per point.

## Results

### Highly Clustered Population of *Listeria monocytogenes* Disclosed by WGS Analysis

**Figure 1:**
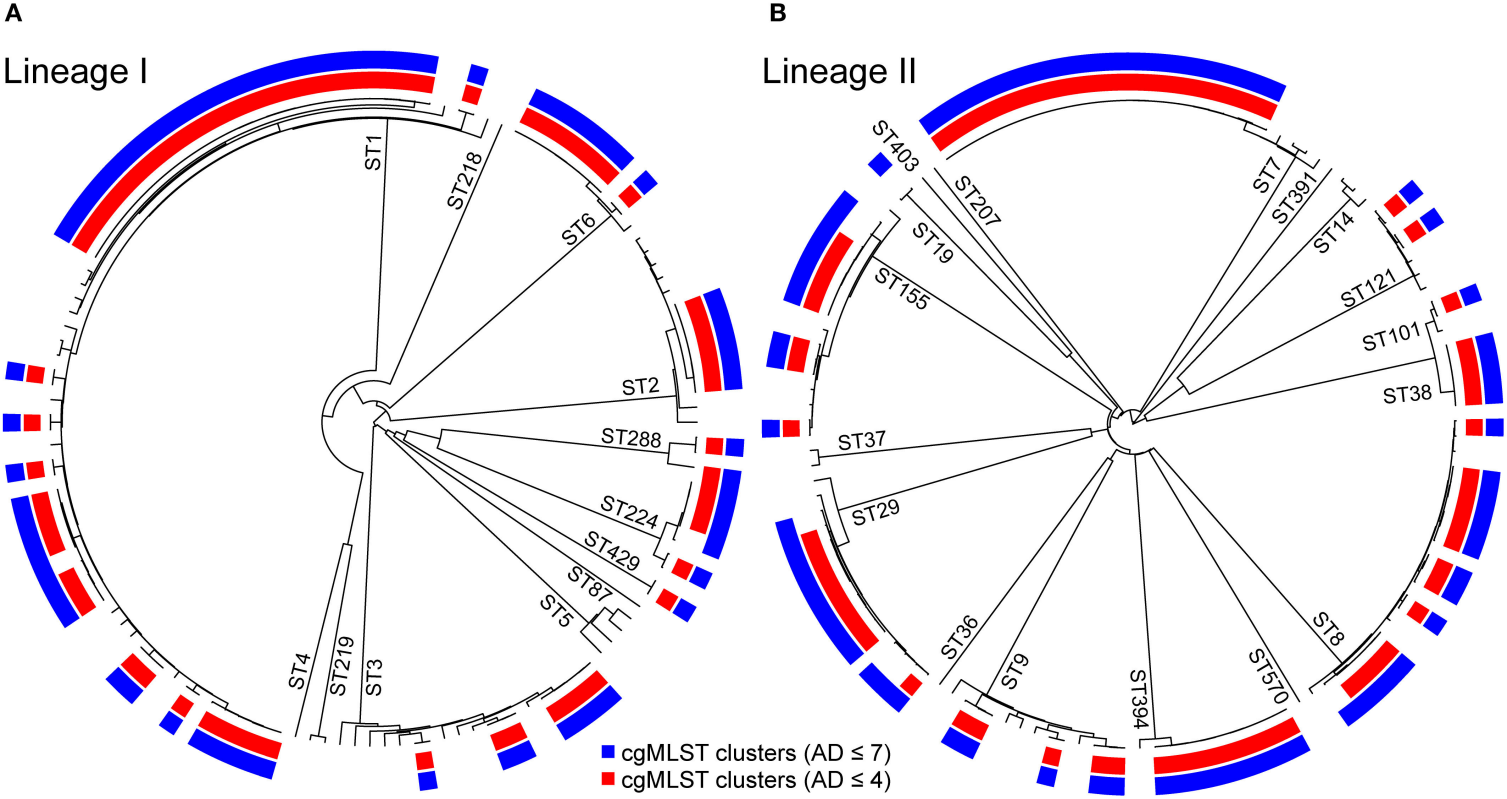
Clustering of 268 *L. monocytogenes* lineage I, panel **(A)**, and lineage II, panel **(B)** isolates from 2012 to 2018 clinical cases in Emilia-Romagna Region based on single-linkage analysis of the cgMLST profiles. The sequence types (STs) are labeled on the branches. The color bars indicate the clusters obtained using cgMLST allelic difference (AD) with threshold AD ≤ 7 (in blue) and AD ≤ 4 (in red).

**Figure 2:**
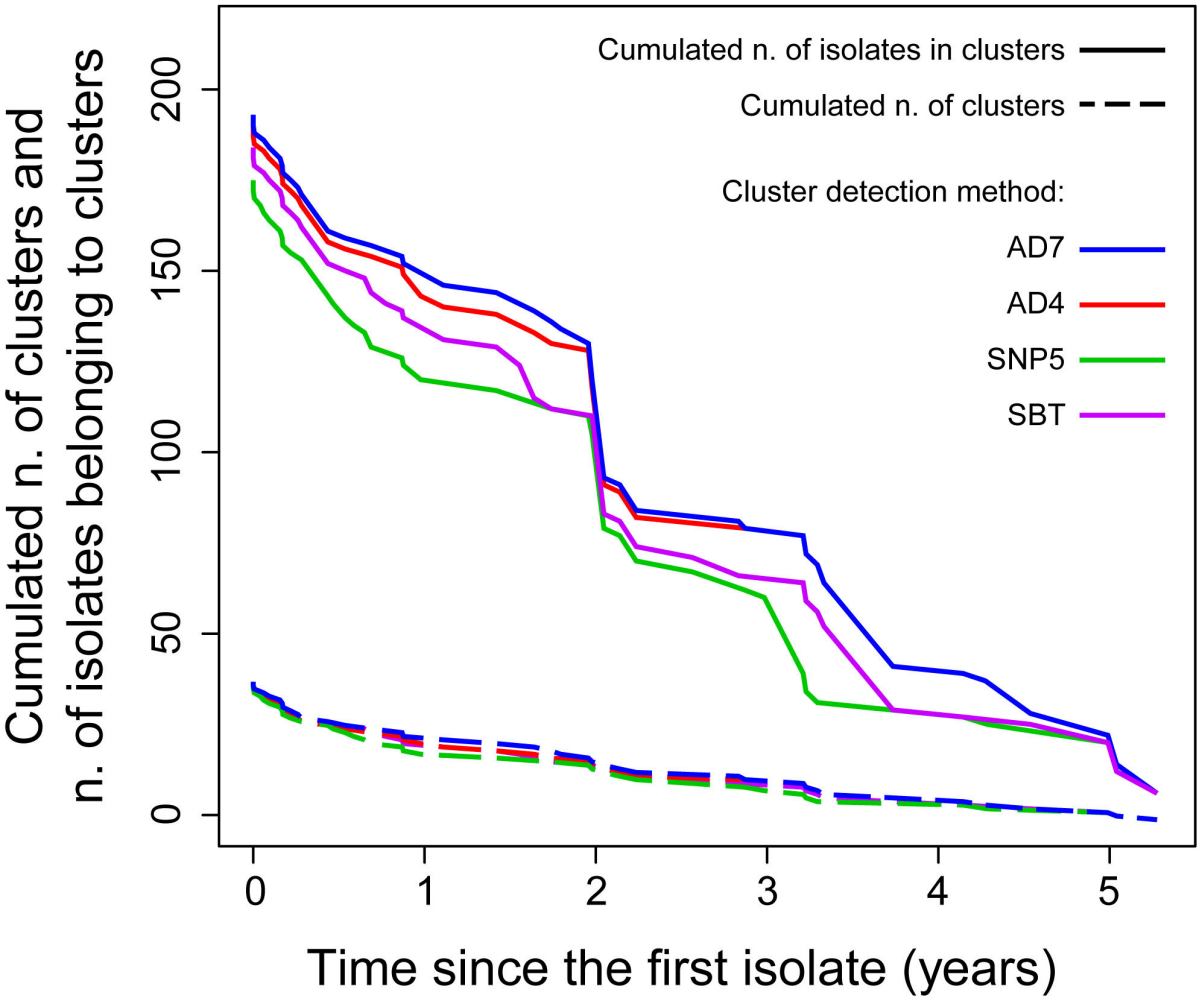
Cumulated number of clusters (solid lines) and cumulated number of isolates belonging to clusters (dashed lines) obtained with different detection methods as functions of the cluster durations (in years).

**Figure 3:**
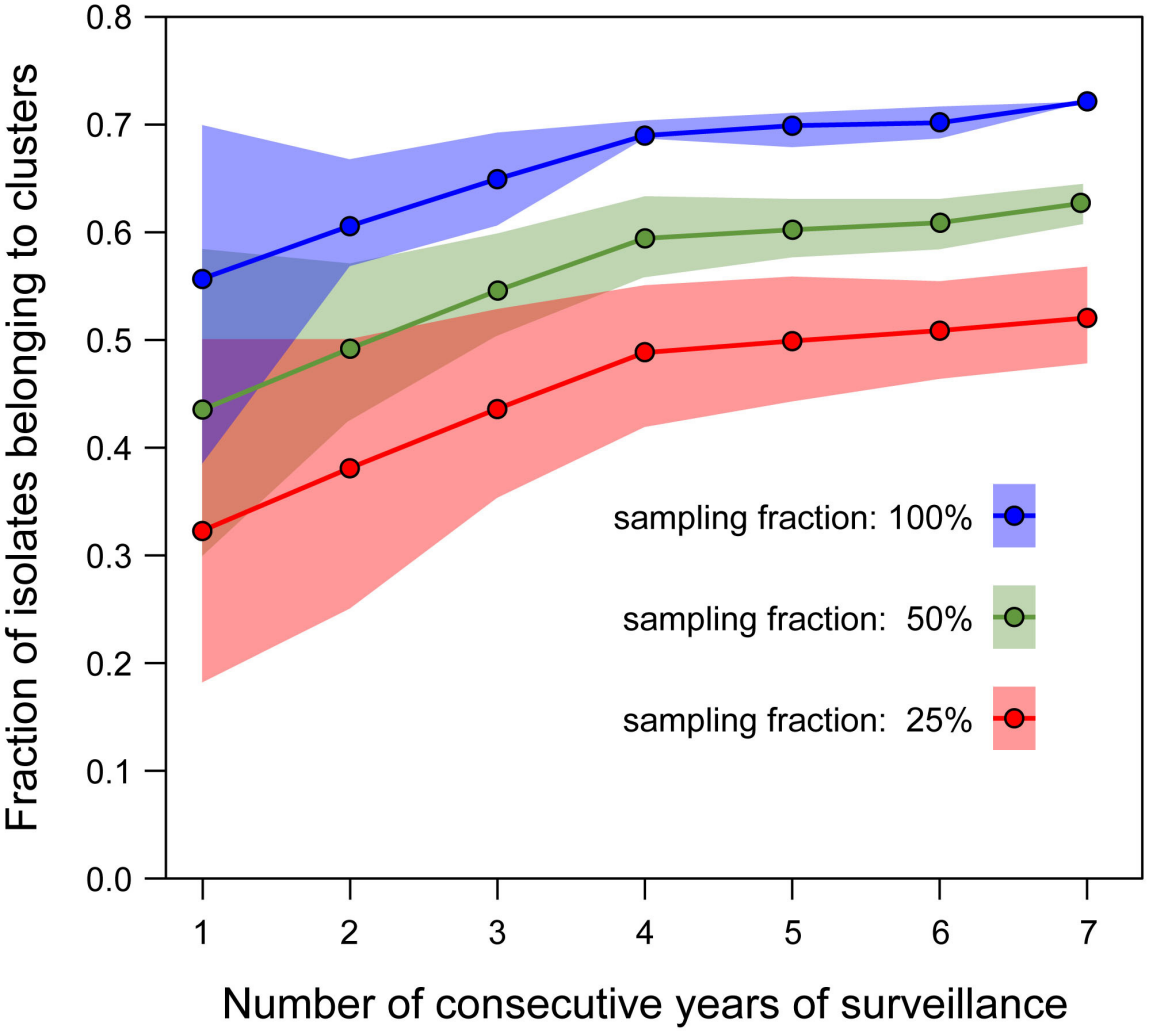
Rarefaction curves estimating the proportion of isolates belonging to clusters (defined using AD7-cgMLST threshold) as a function of the number of consecutive years of surveillance included in the analysis. Each curve corresponds to the analysis performed on a different fraction of the available isolates (blue: 100%, green: 50%, red: 25%). Estimations for all rarefaction curves were performed with 1,000 random samples of the isolates dataset per point. The dots represent the median values, the shaded areas represent the interquartile range.

The isolates were distributed across phylogenetic lineages as follows: 135/268 were lineage I, while 133/268 were lineage II. The population structure of phylogenetic lineage I, panel (A), and II, panel (B), of *L. monocytogenes* in Emilia-Romagna is shown in Figure 1. Twenty-seven CCs (11 in lineage I and 16 in lineage II) and 29 STs (12 in lineage I and 17 in lineage II) were identified by seven-loci MLST, showing that the population is divided into clearly distinct phylogenetic groups. The distribution of isolates across the STs is skew, with the six most prevalent STs (ST1, ST7, ST8, ST3, ST155, ST29) accounting for about 64% of total isolates. Overall cgMLST- and SNP-based analyses show the presence of several clusters in the population, 38–39 depending on the detection method (Figure 2), with 7–11 of the clusters including more than 5 isolates, and the largest consisting of 26–27 isolates. The proportion of cases belonging to clusters ranges from 69 to 72% (when estimated through cgMLST criteria) and it ranges from 66 to 71% (when estimated through SNP-based criteria)—indicating that most cases of listeriosis in ER could be attributed to possible outbreaks—with 18–23 clusters lasting more than 1 year (up to five) and 117–146 (43–54%) of isolates belong to multi-year clusters. The performance of methods is compared in Figure 2. The maximum number of clusters and the largest proportion of cluster-associated cases were identified by AD7-cgMLST while SNP-based detection with cut-off ≤ 5 SNPs (SNP5) identified the minimum, providing maximum discrimination. The results of the rarefaction analysis, using AD7 criterium for cluster definition, are summarized in Figure 3 (see Figure S1 for the analysis with AD4). The analyses showed that the proportion of isolates belonging to clusters crucially depends—as expected—on the fraction of isolates included, but also on the number of consecutive years of surveillance considered. In particular, Figure 3 shows that this proportion decreases for datasets shorter than 4 years, with a drop of more than 10% for datasets of 2 years (or less) compared with datasets of four (or more) years, regardless of the fraction of isolates included.

All 108 genomes of *L. monocytogenes* publicly available from the other Italian regions collected in the 2012–2018 period, representing about 10% of the 1,061 confirmed invasive listeriosis cases in Italy (9, 10) were jointly analyzed with the ER isolates through SNP-based procedures with the aim of: (i) investigating the possible extension of the clusters beyond the borders of ER; and (ii) verifying whether a larger geographical scale leads to the detection of additional clusters that include ER cases. Actually, nine of the clusters detected within ER included isolates from neighboring regions and two additional small clusters (with two and four isolates, respectively) were detected upon extension of the geographical scale. (See the SNP-based phylogenetic trees in Figures S2–S12).

### Complexity of WGS Interpretation Illustrated by Analysis of Lineage II-Predominant ST7

**Figure 4:**
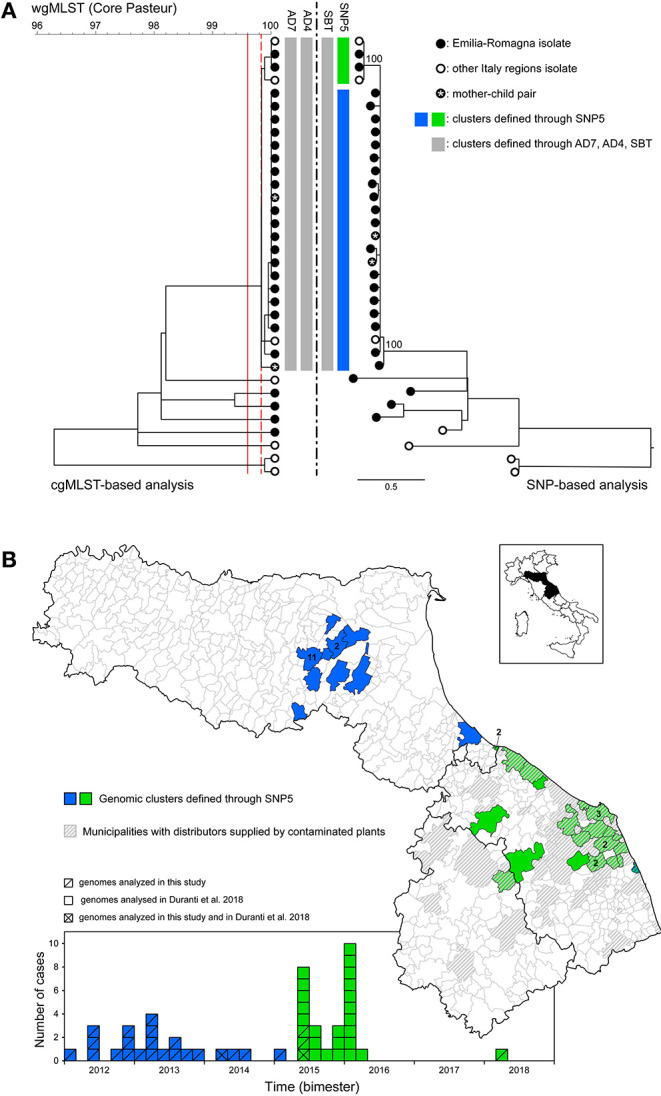
Investigation of the lineage II-predominant sequence type (ST7). **(A)** Comparison between the clustering based on cgMLST (left) and the SNP-based phylogeny (right) including human isolates from Emilia-Romagna (black dots) and from other Italian neighboring regions (white dots). The vertical gray bars indicate the unique genomic cluster detected within ST7 based on cgMLST with thresholds AD7 (4) and AD4 (8) and based on SNPs—with threshold SBT, as defined in (6). The blue and green bars indicate the two genomic clusters detected within ST7 based on the threshold proposed by Dallman et al. (24) (SNP5). Vertical solid and dashed red lines represent the cgMLST threshold AD7 and AD4, respectively. Numbers at nodes in the SNP-based analysis represent percentage bootstrap values and the scale bar unit is the nucleotide substitutions per site. **(B)** Geographical (upper panel) and temporal (bottom panel) distribution of the isolates belonging to the blue and green clusters detected using the threshold proposed by Dallman et al. (24) (SNP5). Gray striped areas indicate the municipalities with food distributors supplied by the contaminated plants identified following epidemiological investigations, as reported in (25).

About 20% of ER lineage II isolates (*n* = 27) belonged to ST7 (Figure 1), featuring a main cgMLST cluster of 23 cases (Figure 4). The cluster included, among the others, a mother-child pair of isolates and three additional genomes from a neighboring region. This mother-child pair of isolates clearly represents a duplicate of the same strain and as such the pair should be included as a single isolate in the analysis for cluster detection in order not to artificially generate false clusters. Nevertheless, in this study both isolates of the pair were included with the specific aim of testing the ability of the different approaches to assign the pair to the same cluster, as expected. Actually, all cgMLST and SNP-based methods placed the mother-child pair in the same cluster (see Figure 4A). In the specific context of the study, the inclusion of this pair of isolates did not result in an artificial increase of the number of detected clusters as the pair was part of the main ST7 cluster together with several other isolates. With regard to this cluster, while the SNP-based method SBT defined by Pigthling et al. (6) provided the same clustering as cgMLST methods, the SNP-based method derived from (24) divided the same isolates in two distinct clusters (blue and green in Figure 4A). Actually, available epidemiological evidence does support the existence of two distinct clusters with independent origin. Firstly, the two extra-ER genomes of the green cluster were part of a confirmed outbreak of 25 cases affecting two neighboring regions caused by the consumption of pork products as investigated by Duranti et al. (25). Therefore, the two ER cases of the green cluster very likely belonged to that outbreak also considering their closeness to the outbreak area (Figure 4B). Concomitantly, during the investigation of the outbreak, those authors also detected cases belonging to the blue cluster on their territory but could not demonstrate epidemiological association of those cases with the food producers implicated in the outbreak and eventually classified the cases as not belonging to the outbreak. Secondly, the two clusters are geographically and temporally segregated (Figure 4B). Thirdly, the trade area of the responsible food overlaps the outbreak territory (green cases) but not the area of the blue cluster (Figure 4B). The independence of the two clusters is further confirmed by the presence of a 31 kb prophage integrated in the green cluster genomes of ER and extra-ER origin as well as in the isolates from the pork products, but missing in the blue cluster genomes (13).

Altogether, these findings would suggest the existence of independent sources for the green and blue clusters; nevertheless, further understanding of the relationship between the two clusters emerges from a more in-depth analysis of the genomic information available. Their overall genetic similarity is considerable, with pairwise difference of only 9–12 SNPs (min-max); moreover, the phylogenetic tree topology shows that the green cluster originates from the blue cluster as a sub-clade (Figure 4A) and, consistently with this observation, the green cases occurred after the blue ones. Overall, these data indicate the possible existence of a recent shared origin for the two clusters represented by a source of contamination responsible at first of the blue cases and later causing the green cases through the expansion of a sub-clone of *L. monocytogenes* from the original clone. This sub-clone was evidently distributed through the pork products associated with the green outbreak, while the direct vehicle of infection of the blue cases remained undisclosed.

### Use of WGS in Prospective Surveillance

**Figure 5:**
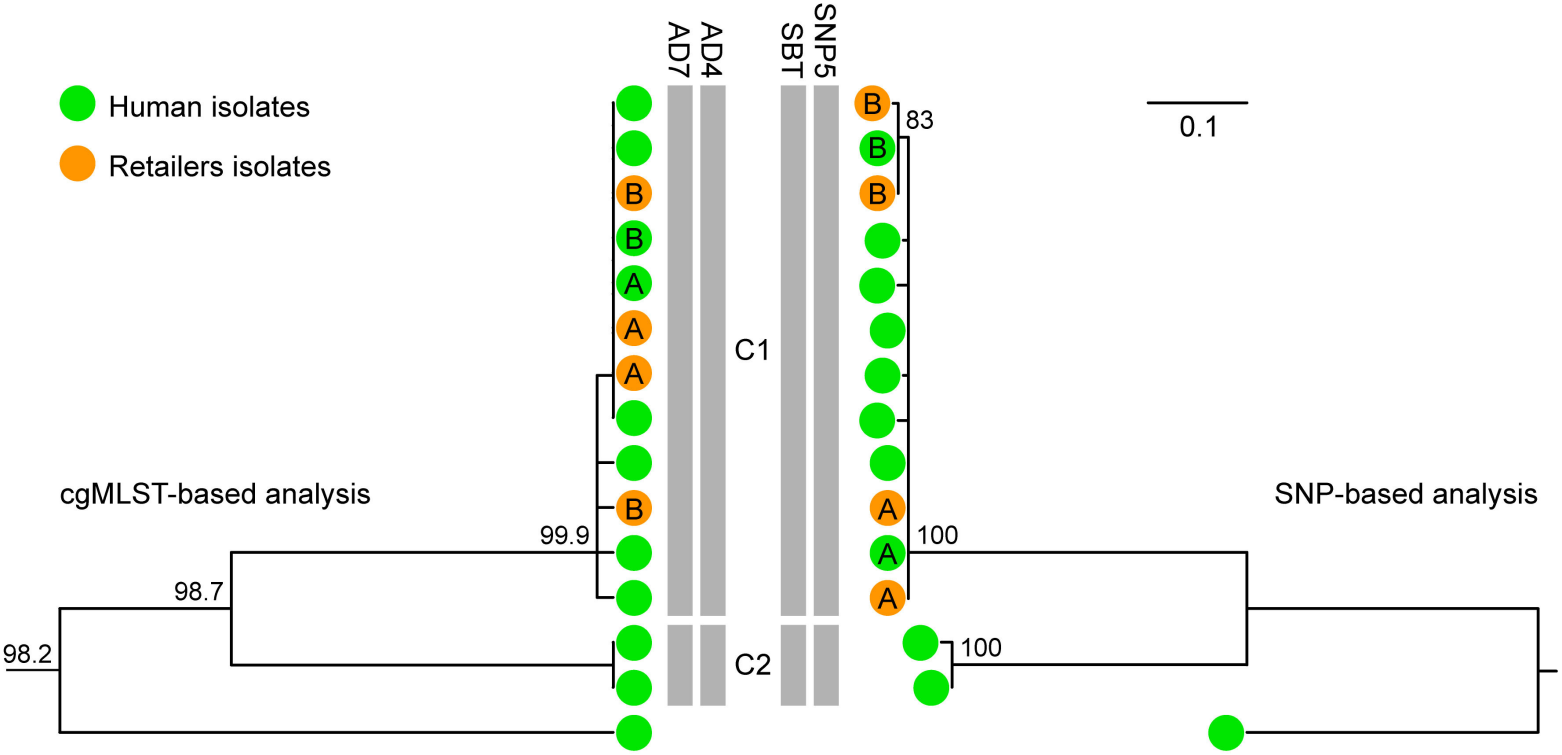
Comparison between the clustering based on cgMLST (left) and the SNP-based phylogeny (right) of the *L. monocytogenes* isolates belonging to ST6 from 2012 to 2018 originated from clinical cases in Emilia-Romagna Region (green dots, 11 isolates) and from environmental samples in two retailers (namely A and B, orange dots, four isolates). Letters on the green dots refer to the isolates from cases epidemiologically linked to retailers A and B, respectively. The vertical gray bars indicate the genomic clusters (namely, C1 and C2) detected within ST6 based on cgMLST with thresholds AD7 (4) and AD4 (8) and based on SNPs with threshold SBT (6) and with threshold SNP5 (24). Numbers at nodes in the cgMLST-based analysis represent the similarity values derived from single-linkage clustering. Numbers at nodes in the SNP-based analysis represent percentage bootstrap values and the scale bar unit is the nucleotide substitutions per site.

WGS typing was integrated in the prospective surveillance of invasive listeriosis in Emilia-Romagna in 2018. Forty-six cases were notified to the surveillance system in 2018 (1.03 cases per 100,000 population). The cgMLST-based surveillance identified nine new genomic clusters, which included 21 isolates, while another 17 isolates were assigned to six previously detected clusters. Two of these clusters, which included four or more 2018 isolates, underwent further epidemiological and microbiological investigation to identify the source of infection. The choice to further investigate these two clusters was based on the evidence produced by Rounds et al. (26, 27) that genetic clusters of ≥4 isolates from foodborne infections were more likely to be epidemiologically confirmed than clusters with less isolates. The investigated clusters belonged to ST224 (with four 2018 cases) and ST6 (with six 2018 cases). No suspected source was identified for ST224 cluster. On the other hand, three cases included in the ST6 cluster were linked, through food questionnaires, to the consumption of pork products sold in large retail shops located in three different provinces of the region. All six 2018 cases of the ST6 cluster were hospitalized and three died. Environmental swabs collected at the retail shops on equipment used for the pork sale were positive for *L. monocytogenes* belonging to the ST6 cluster in two of the three shops (see cluster C1 in Figure 5). Specifically, isolates matching the ST6 cluster were sampled on a meat slicer and a sausage knife in one of the shops (retailer A) and on a meat slicer and on the surface of the pork counter in the other (retailer B). Figure 5 shows that both the cgMLST-based and the SNP-based metrics assigned the 2018 cases to cluster C1 along with the retailers' isolates. However, despite the overall high genetic similarity within cluster C1 (pairwise SNP difference: median = 2, range = 0–5), the SNP-based phylogeny provided better resolution than cgMLST by differentiating (although with bootstrap value = 83) the isolates linked to retailer B, both clinical and environmental, from the others within cluster C1 (see Figure 5). The trace-back to a specific food source shared by the retailers was not conclusive.

In 2018, the regional surveillance system evaluated two ECDC urgent inquiries upon request of the Italian Operational Contact Point for listeriosis, UI-426 (28), and UI-444 (29), concerning multi-country outbreaks in Europe. No sequences fulfilling the matching criteria set in the inquiries were present in the regional database.

## Discussion

The results of the study reveal that the population of *Listeria monocytogenes* from humans of ER is predominantly structured in clusters of isolates similar enough to be indicative of several possible listeriosis outbreaks over the 7 years of observation. Indeed, between 66 and 72% of the isolates, depending on the method adopted, belong to genomic clusters. This finding contrasts with the evidence that no outbreaks were detected in ER before the implementation of WGS-based surveillance, essentially due to the scattering in time and space of the cases that were consequently classified as sporadic instead of outbreak-associated. This highlights the critical importance of WGS methods to support *L. monocytogenes* surveillance for outbreak identification. Supportive evidence of this was the first identification of an outbreak by *L. monocytogenes* in ER in the first year of adoption of prospective WGS surveillance in 2018. While this is widely recognized, the cluster-associated proportion of cases that we observed was higher than those reported through cgMLST methods by Moura et al. (23), 40% (AD7 threshold), and Van Walle et al. (8), 45% (AD4) and 52% (AD7 threshold), who investigated the population structure of *L. monocytogenes* in France and Europe, respectively. As shown by the rarefaction analysis (see Figure 3), the observed difference with Moura et al. (23) could be attributed to the wider time-window (seven vs. 2 years) and to the higher rate of notified cases (0.91 vs. 0.59 per 100,000 population per year) in our study compared to the French one. Specifically, the rarefaction analysis predicts (both for AD4 and AD7) a drop of about 13% in the percentage of cluster-associated isolates following a reduction of the time-window from seven to 2 years. The observed difference with Van Walle et al. (8) could be attributed to the higher percentage of confirmed cases undergone WGS included in our study compared to the European one, where <24% of listeriosis cases reported in the European Union over a 6-year time-window were included, as opposed to 95% of the cases reported from Emilia-Romagna. Interestingly, our rarefaction analysis performed on a 6-year time-window including only 25% of our isolates predicts a proportion of cluster-associated isolates strikingly similar to that estimated by Van Walle et al. (8), i.e., 46.5% (with AD4) and 50.8% (with AD7). In agreement with our finding, Van Walle et al. (8) highlighted that the proportion of sporadic cases estimated in their work is likely to be lower for more comprehensive samplings. These findings indicate that a wide time-window and high surveillance intensity (intended as both high notification rate and high WGS-typing rate) represent critical conditions for the sensitive detection of potential listeriosis outbreaks in a WGS-based surveillance. This high rate of WGS-typed isolates can only be achieved through optimal local interoperation of diagnostic laboratories, typing laboratories, and health services which is easier to achieve through distributed systems on the territory than through centralized organizations. At the same time, our results confirmed that outbreaks can extend beyond a single region and even a single country as already observed in Europe (8, 29, 30). This simultaneous need of local-scale interoperation and large-scale cooperation suggests that networks of regional services and laboratories connected to each other in a coordinated organization could represent a proper approach to effective surveillance as a balanced solution to both needs.

Our study highlighted the existence of multi-year clusters, up to 5 year long. This finding reflects the ability of *L. monocytogenes* to persistently colonize food-processing facilities, as already reported (31–33) and has an effect on newly implemented WGS surveillance in its starting stage. In fact, gathering a multi-year retrospective dataset of genomic sequences is essential for reliable cluster detection at the beginning of surveillance when incoming isolates often belong to outbreaks originated in the past.

The performance of four previously validated cgMLST- and SNPs-based methods was evaluated. While generally providing comparable outcomes in cluster detection, cgMLST- and SNPs-based approaches may sometimes differ in assigning some isolates to clusters and in the overall inclusiveness of isolates and resolution of clusters. In particular AD7-cgMLST determined the largest number of clusters and proportion of cluster-associated cases, while SNP-based detection with cut-off ≤ 5 SNPs provided the highest discrimination between clusters. According to these results, AD7-cgMLST appears the best rapid approach (screening tool) to detect putative outbreaks from WGS data with maximum sensibility, also considering its independence from dataset size and consequent scalability to large numbers of isolates and the immediacy to share information on cgMLST data among laboratories. On the other hand, SNP-based analysis on selected and relatively small sub-sets of data appears necessary to refine the analysis and accurately confirm clusters, as indicated by Schurch et al. (34) and Pightling at al. (6). The importance of adopting a multiple approach has clearly emerged in the investigation of the ST7 cluster, where both SNP-based analysis, including SNP differences and phylogenetic tree topology, and the analysis of the accessory genome (a prophage) were needed to elucidate the likely epidemiological dynamics of the cluster.

## Data Availability Statement

The datasets generated for this study can be found in the EBI under Project number PRJEB34036.

## Author Contributions

ES, LB, and SP conceived and design the study. ES, CV, MM, IM, and MR collected the samples and performed the laboratory analysis. ES, LB, and IM analyzed the data. LB and SP drafted the manuscript. All authors revised the manuscript.

## Conflict of Interest

The authors declare that the research was conducted in the absence of any commercial or financial relationships that could be construed as a potential conflict of interest.
