# Supplementary material for: Population Structure of Listeria monocytogenes in Emilia-Romagna (Italy) and Implications on Whole Genome Sequencing Surveillance of Listeriosis
Source: Front Public Health. 2020 Sep 18;8:519293. doi: 10.3389/fpubh.2020.519293 (PMC7531028; doi:10.3389/fpubh.2020.519293)
Supplement: Supplementary file 1 [file Data_Sheet_1.PDF]

APPENDIX A. Supplementary Data

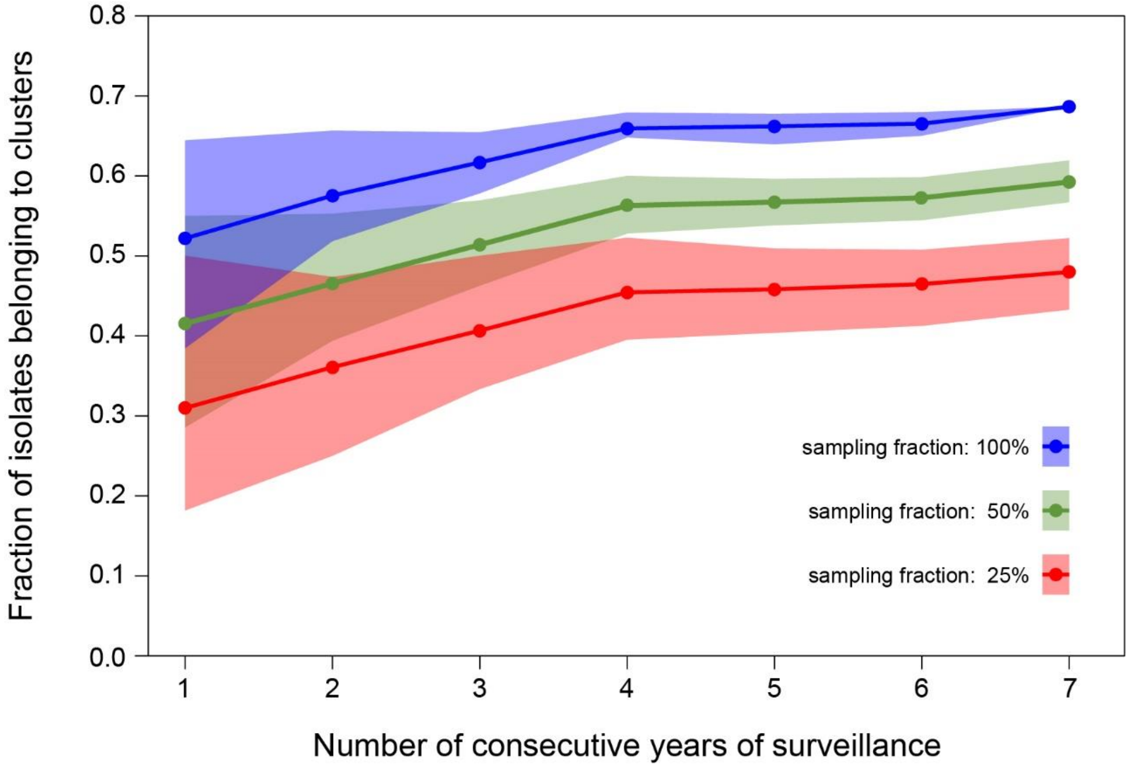

Fig. S1: Rarefaction curves estimating the proportion of isolates belonging to clusters (defined using AD4-cgMLST threshold) as a function of the number of consecutive years of surveillance included in the analysis. Each curve corresponds to the analysis performed on a different fraction of the available isolates (blue: 100%, green: 50%, red: 25%). Estimations for all rarefaction curves were performed with 1,000 random samples of the isolates dataset per point. The dots represent the median values, the shaded areas represent the interquartile range.

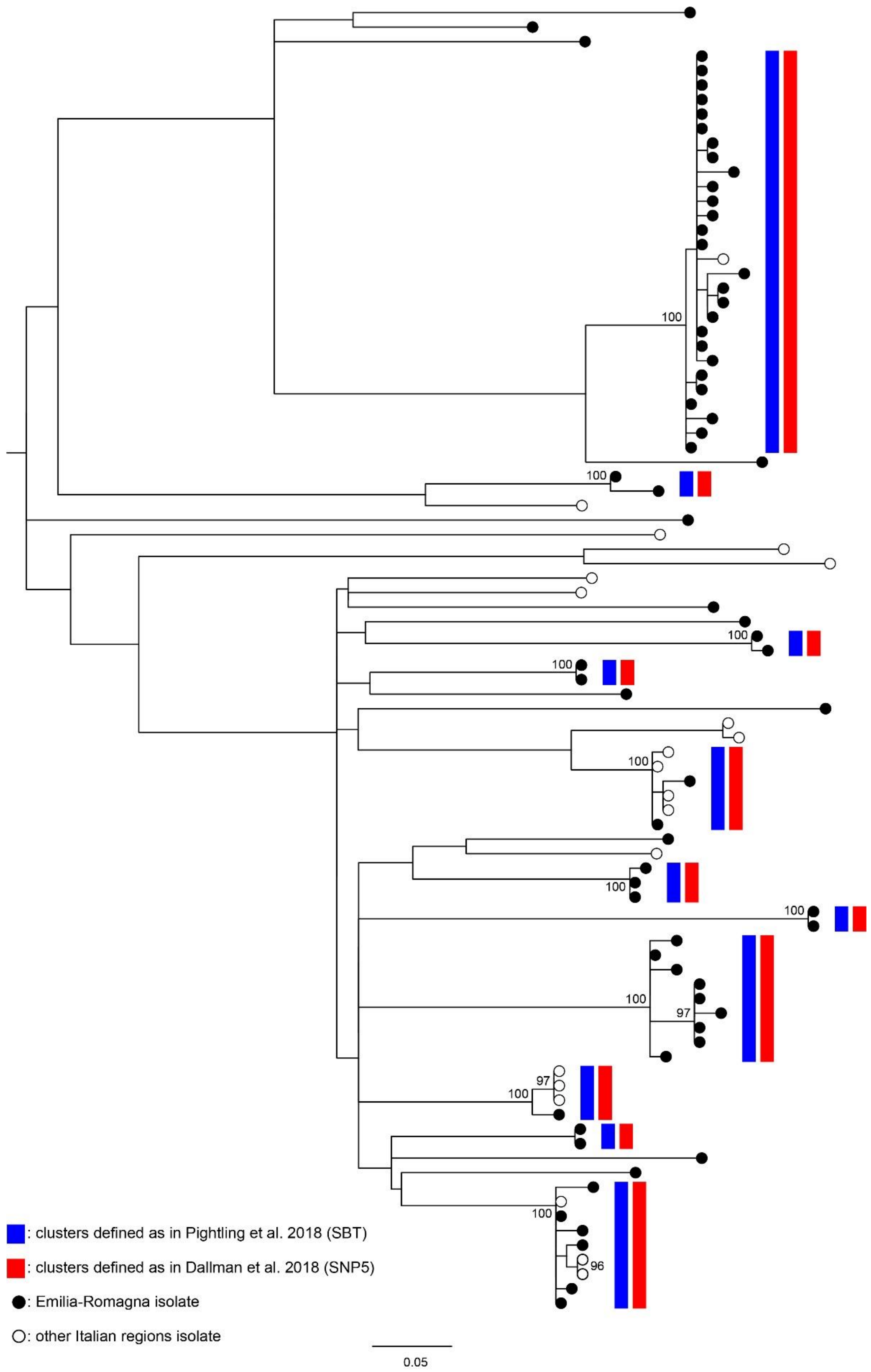

Fig. S2: the SNP-based phylogeny including human isolates from Emilia-Romagna (black dots) and from other Italian neighboring regions (white dots) for isolates belonging to ST1. The vertical bars indicate the genomic clusters detected within ST1 based on the thresholds proposed in Pightling et al. 2018 (SBT, in blue) and in Dallman et al. 2018 (SNP5, in red). Numbers at nodes represent bootstrap values in supported clusters (i.e. ≥90).

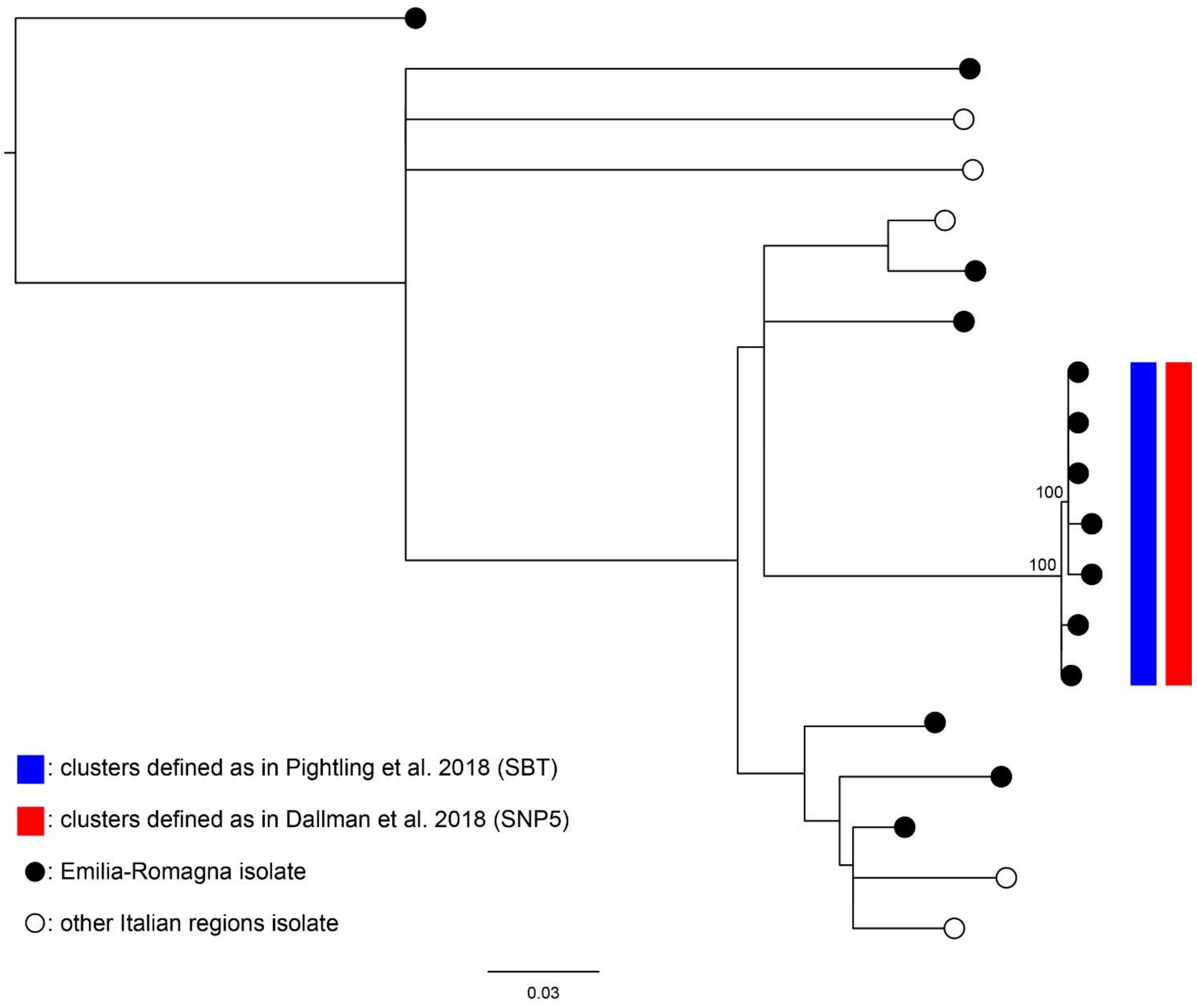

Fig. S3: the SNP-based phylogeny including human isolates from Emilia-Romagna (black dots) and from other Italian neighboring regions (white dots) for isolates belonging to ST2. The vertical bars indicate the genomic clusters detected within ST2 based on the thresholds proposed in Pightling et al. 2018 (SBT, in blue) and in Dallman et al. 2018 (SNP5, in red). Numbers at nodes represent bootstrap values in supported clusters (i.e. ≥90).

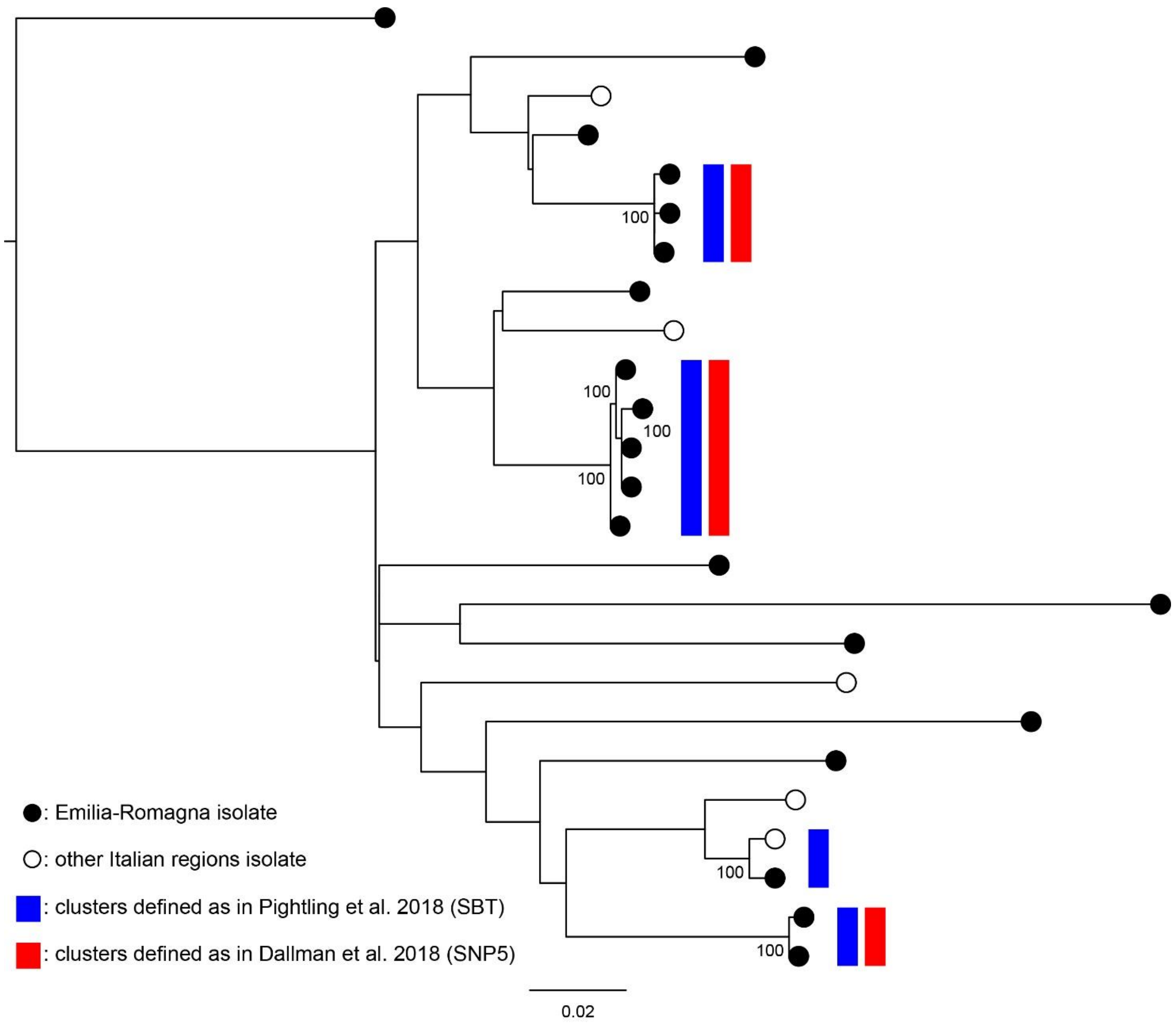

Fig. S4: the SNP-based phylogeny including human isolates from Emilia-Romagna (black dots) and from other Italian neighboring regions (white dots) for isolates belonging to ST3. The vertical bars indicate the genomic clusters detected within ST3 based on the thresholds proposed in Pightling et al. 2018 (SBT, in blue) and in Dallman et al. 2018 (SNP5, in red). Numbers at nodes represent bootstrap values in supported clusters (i.e. ≥90).

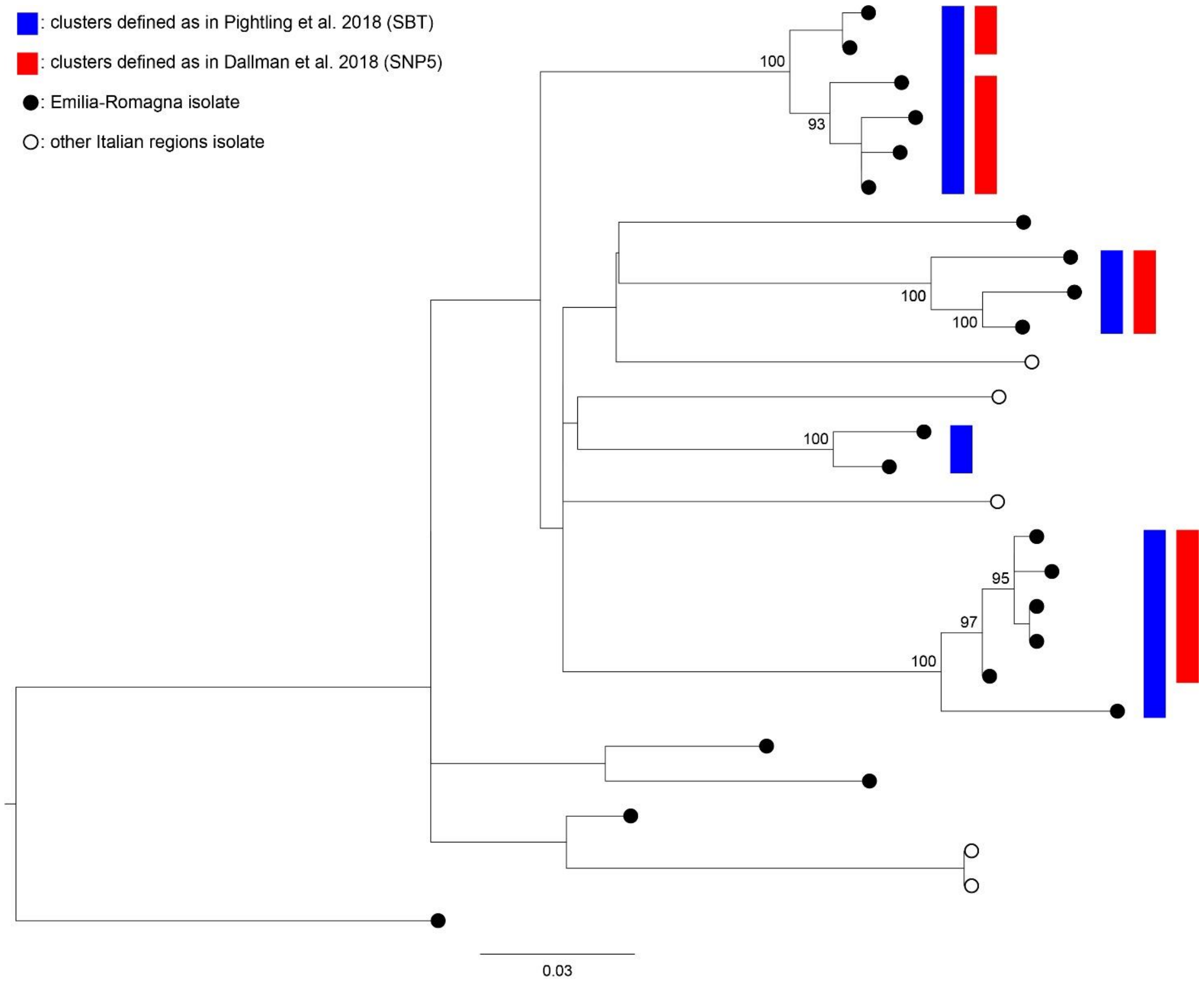

Fig. S5: the SNP-based phylogeny including human isolates from Emilia-Romagna (black dots) and from other Italian neighboring regions (white dots) for isolates belonging to ST8. The vertical bars indicate the genomic clusters detected within ST8 based on the thresholds proposed in Pightling et al. 2018 (SBT, in blue) and in Dallman et al. 2018 (SNP5, in red). Numbers at nodes represent bootstrap values in supported clusters (i.e. ≥90).

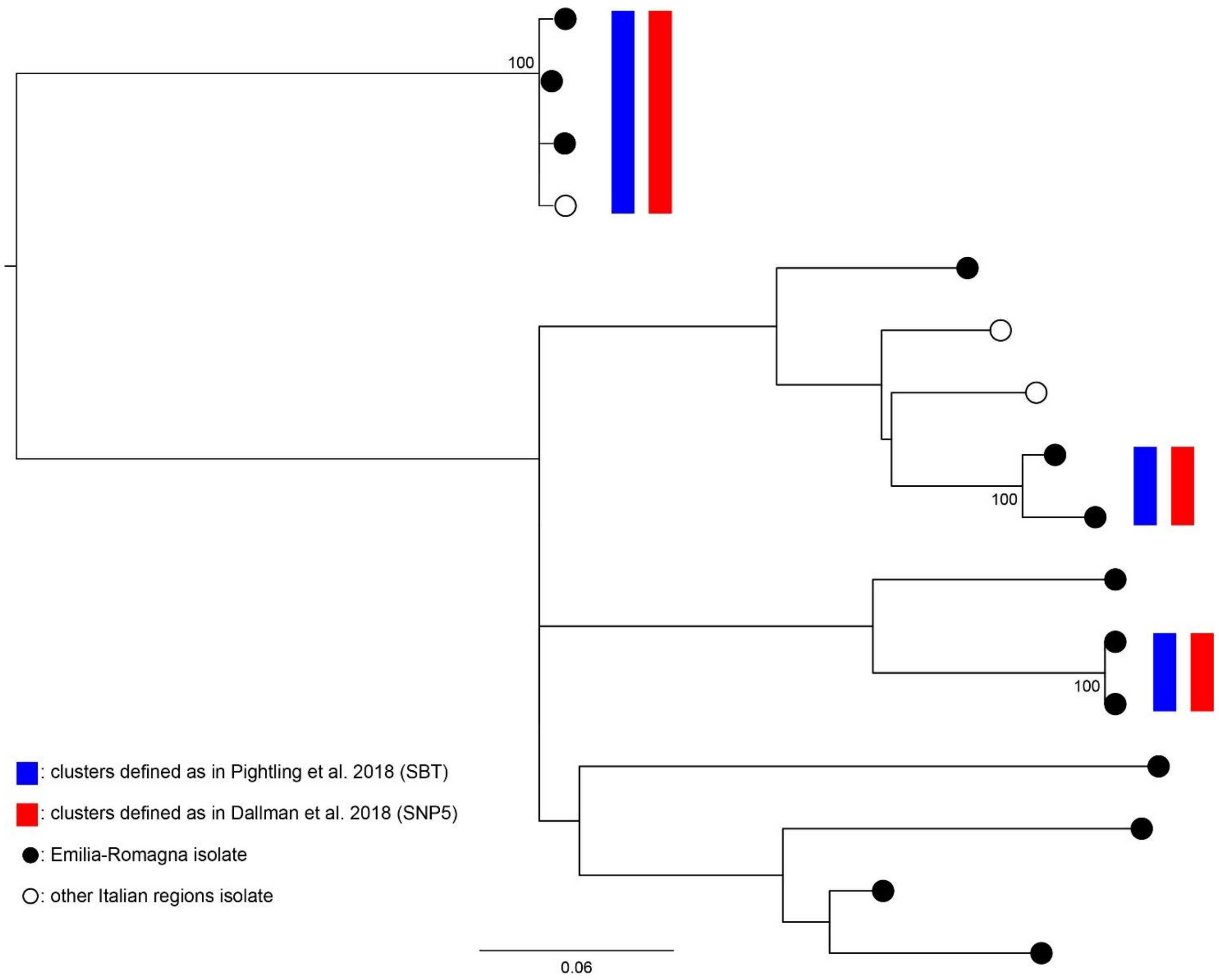

Fig. S6: the SNP-based phylogeny including human isolates from Emilia-Romagna (black dots) and from other Italian neighboring regions (white dots) for isolates belonging to ST9. The vertical bars indicate the genomic clusters detected within ST9 based on the thresholds proposed in Pightling et al. 2018 (SBT, in blue) and in Dallman et al. 2018 (SNP5, in red). Numbers at nodes represent bootstrap values in supported clusters (i.e. ≥90).

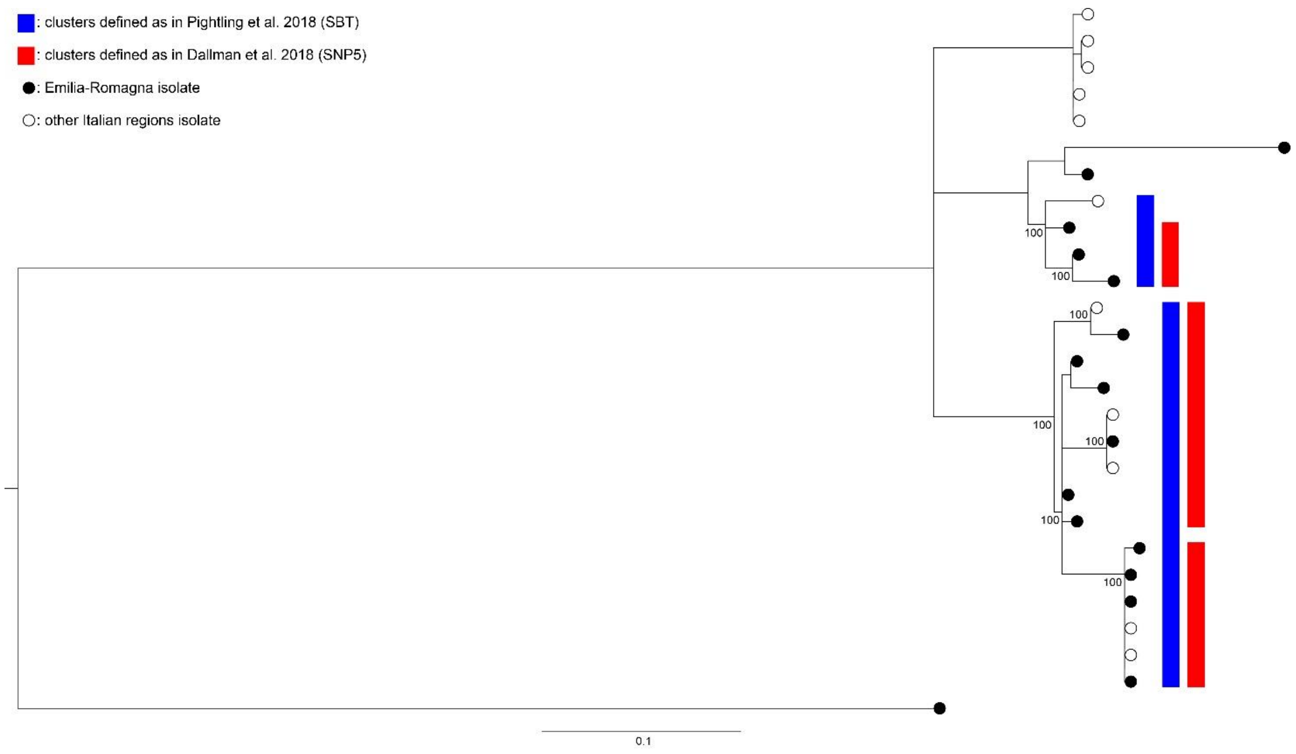

Fig. S7: the SNP-based phylogeny including human isolates from Emilia-Romagna (black dots) and from other Italian neighboring regions (white dots) for isolates belonging to ST29. The vertical bars indicate the genomic clusters detected within ST29 based on the thresholds proposed in Pightling et al. 2018 (SBT, in blue) and in Dallman et al. 2018 (SNP5, in red). Numbers at nodes represent bootstrap values in supported clusters (i.e. ≥90).

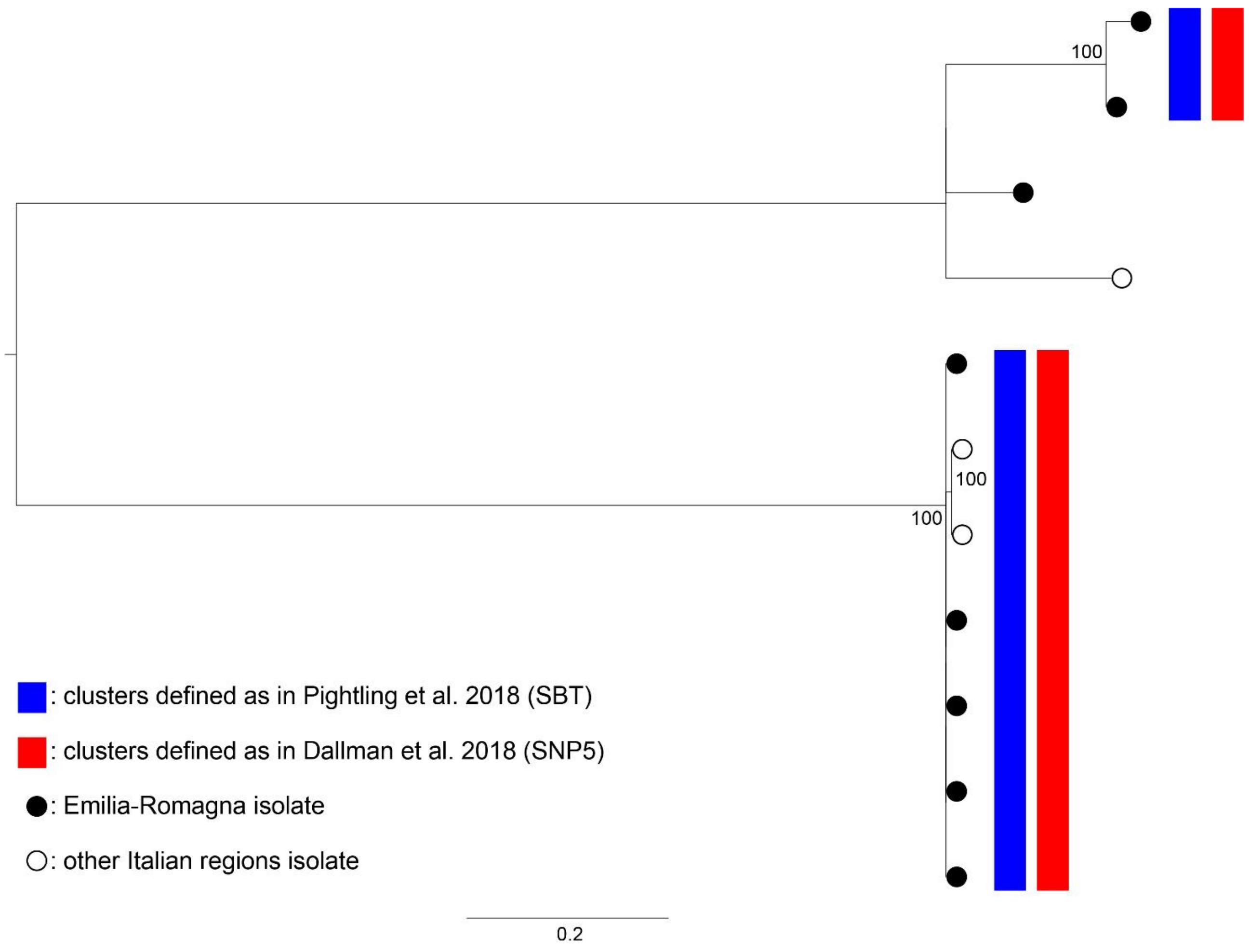

Fig. S8: the SNP-based phylogeny including human isolates from Emilia-Romagna (black dots) and from other Italian neighboring regions (white dots) for isolates belonging to CC101 (including ST38 and ST101). The vertical bars indicate the genomic clusters detected within CC101 based on the thresholds proposed in Pightling et al. 2018 (SBT, in blue) and in Dallman et al. 2018 (SNP5, in red). Numbers at nodes represent bootstrap values in supported clusters (i.e. ≥90).

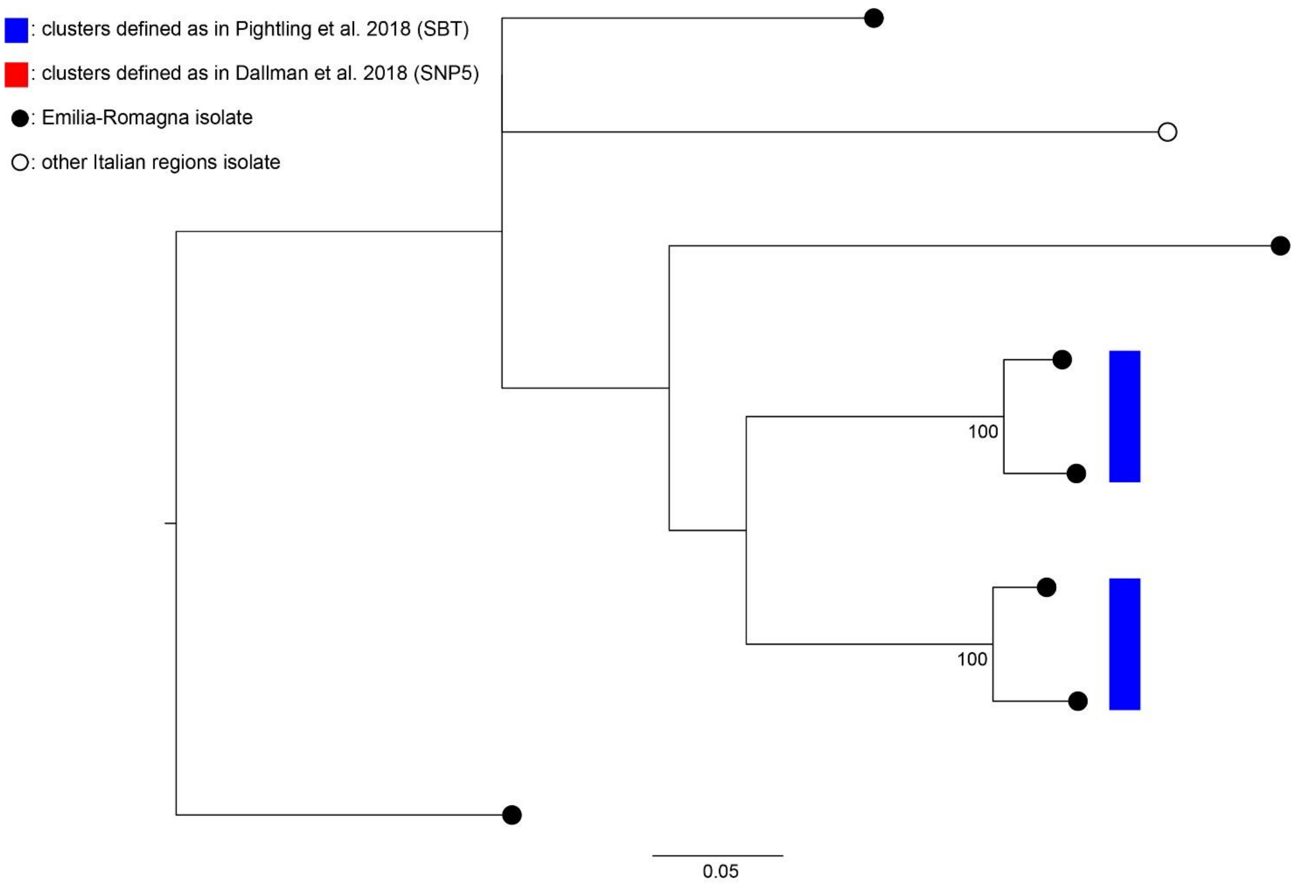

Fig. S9: the SNP-based phylogeny including human isolates from Emilia-Romagna (black dots) and from other Italian neighboring regions (white dots) for isolates belonging to ST121. The vertical bars indicate the genomic clusters detected within ST121 based on the thresholds proposed in Pightling et al. 2018 (SBT, in blue) and in Dallman et al. 2018 (SNP5, in red). Numbers at nodes represent bootstrap values in supported clusters (i.e. ≥90).

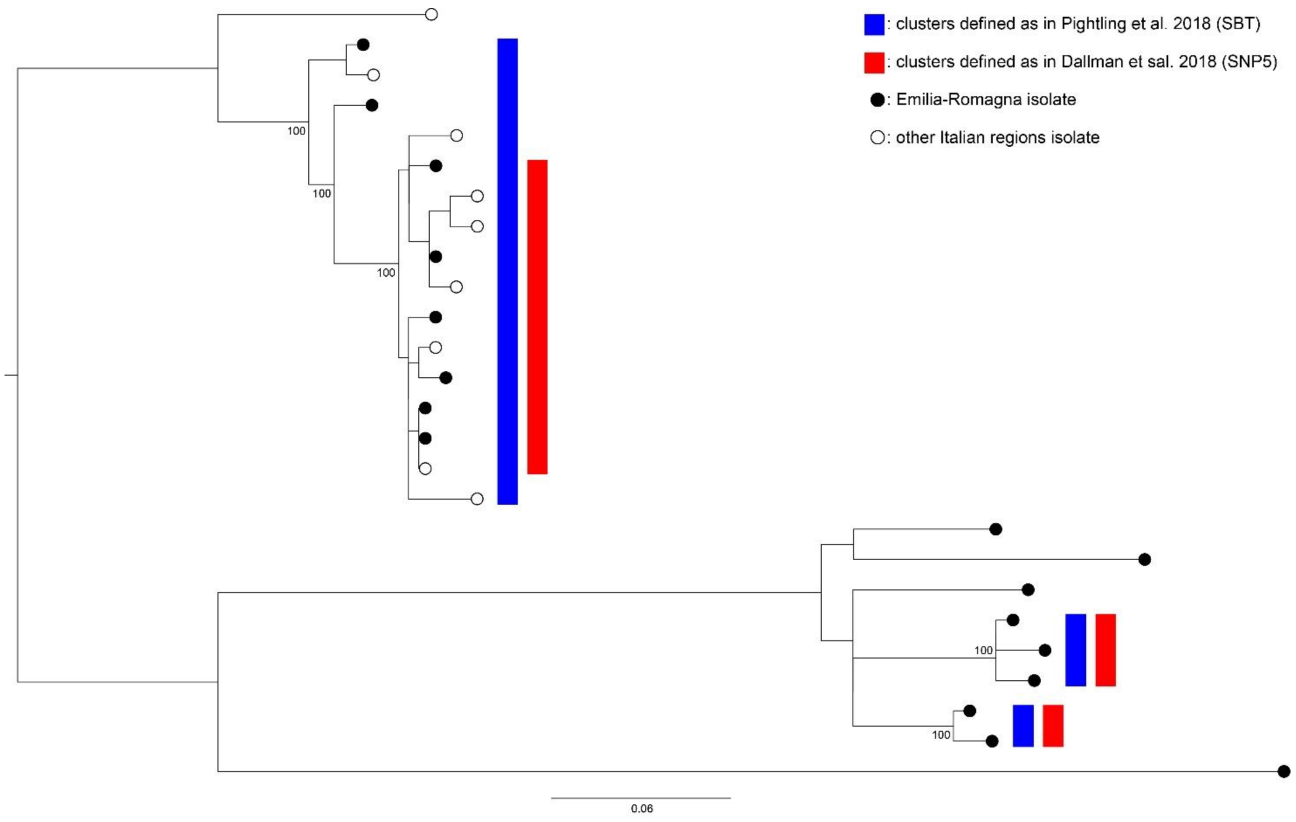

Fig. S10: the SNP-based phylogeny including human isolates from Emilia-Romagna (black dots) and from other Italian neighboring regions (white dots) for isolates belonging to ST155. The vertical bars indicate the genomic clusters detected within ST155 based on the thresholds proposed in Pightling et al. 2018 (SBT, in blue) and in Dallman et al. 2018 (SNP5, in red). Numbers at nodes represent bootstrap values in supported clusters (i.e. ≥90).

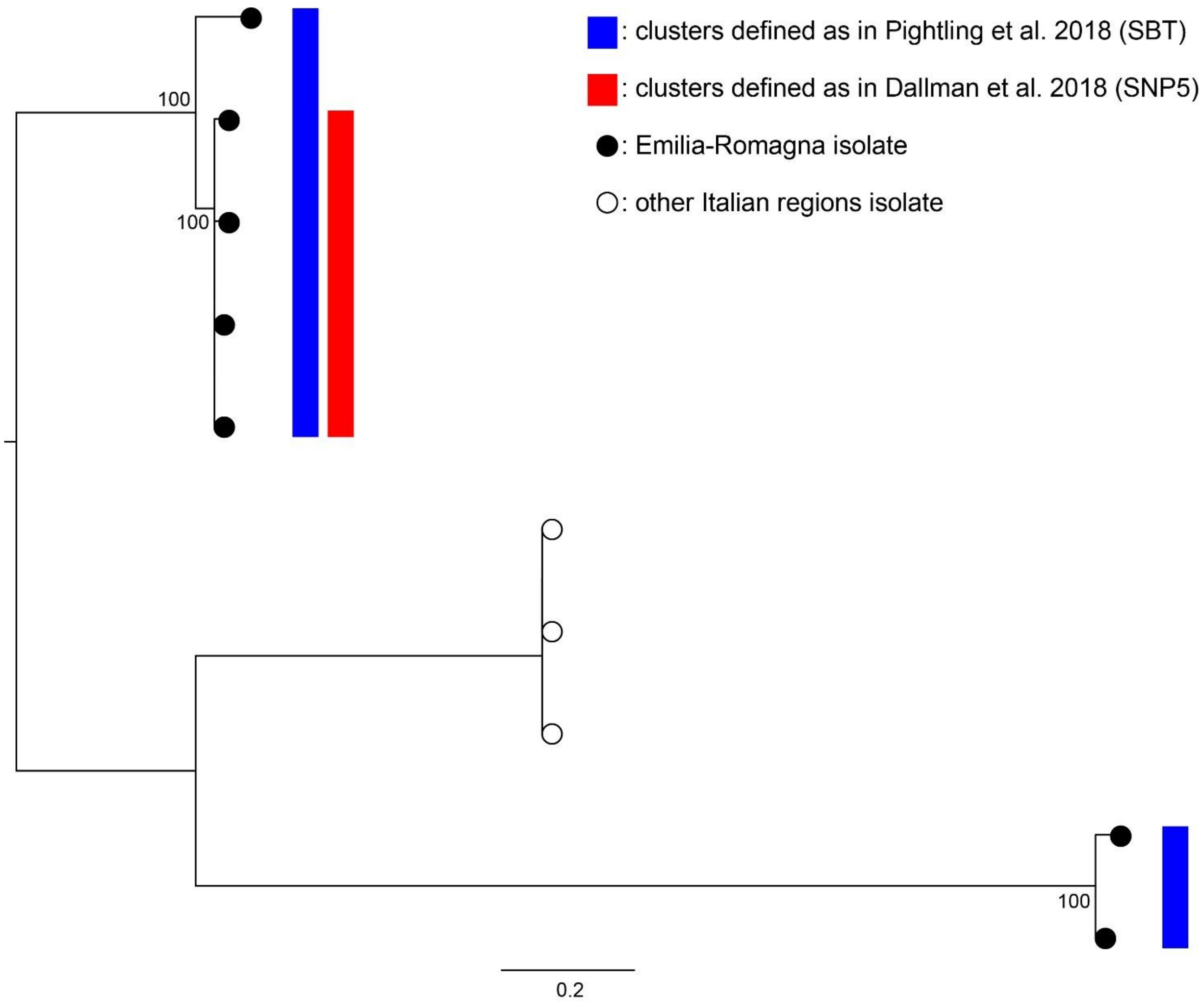

Fig. S11: the SNP-based phylogeny including human isolates from Emilia-Romagna (black dots) and from other Italian neighboring regions (white dots) for isolates belonging to ST224. The vertical bars indicate the genomic clusters detected within ST224 based on the thresholds proposed in Pightling et al. 2018 (SBT, in blue) and in Dallman et al. 2018 (SNP5, in red). Numbers at nodes represent bootstrap values in supported clusters (i.e. ≥90).

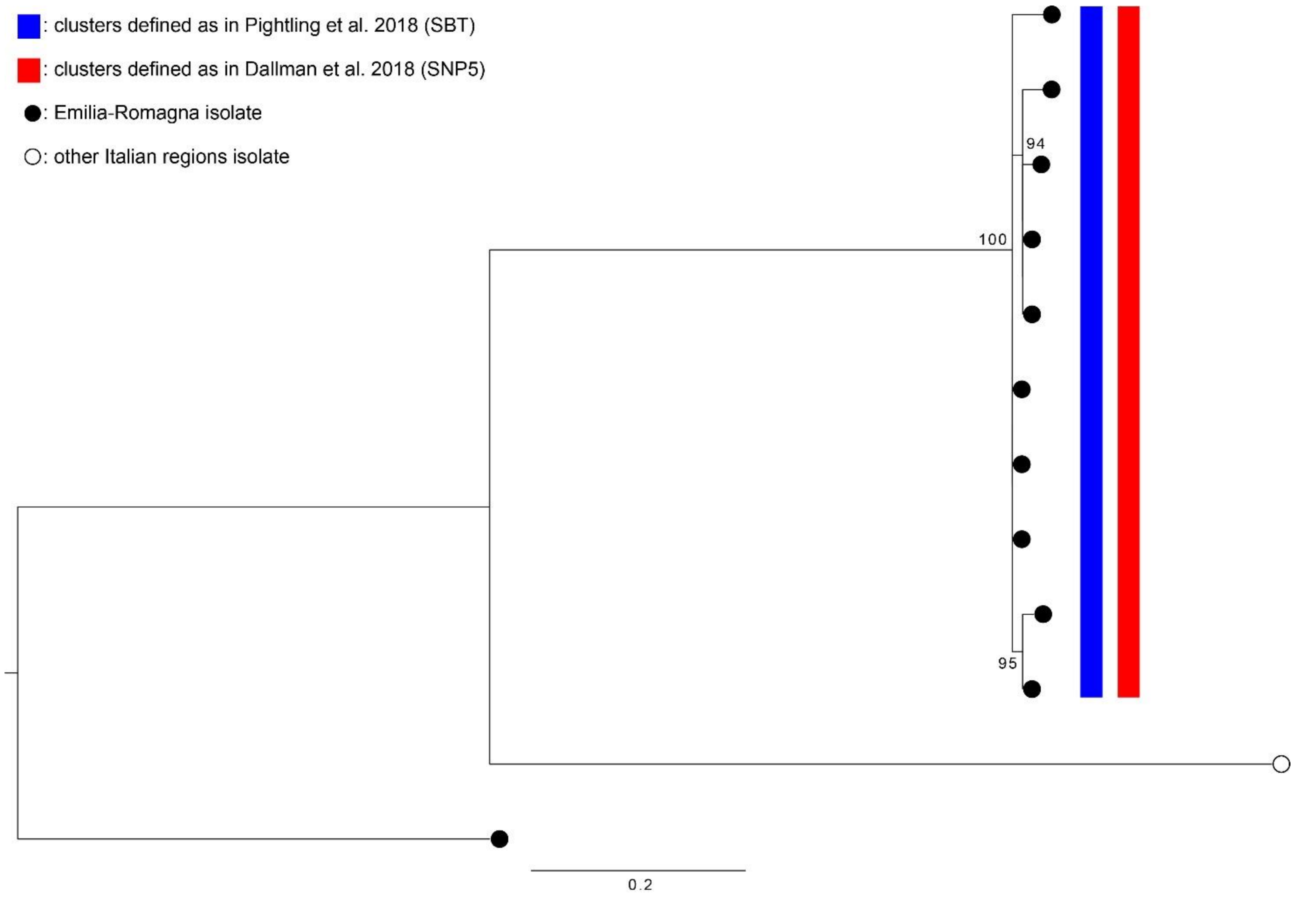

Fig. S12: the SNP-based phylogeny including human isolates from Emilia-Romagna (black dots) and from other Italian neighboring regions (white dots) for isolates belonging to ST394. The vertical bars indicate the genomic clusters detected within ST394 based on the thresholds proposed in Pightling et al. 2018 (SBT, in blue) and in Dallman et al. 2018 (SNP5, in red). Numbers at nodes represent bootstrap values in supported clusters (i.e. ≥90).
